# Temperature Sensor Based on Side-Polished Fiber SPR Device Coated with Polymer

**DOI:** 10.3390/s19194063

**Published:** 2019-09-20

**Authors:** Shuhui Liu, Shaoqing Cao, Zhe Zhang, Ying Wang, Changrui Liao, Yiping Wang

**Affiliations:** Key Laboratory of Optoelectronic Devices and Systems of Ministry of Education and Guangdong Province, College of Optoelectronic Engineering, Shenzhen University, Shenzhen 518060, Chinaypwang@szu.edu.cn (Y.W.)

**Keywords:** optical fiber sensor, surface plasmon resonance, temperature sensor, refractive index

## Abstract

A highly sensitive temperature sensor based on surface plasmon resonance (SPR) of a side-polished single mode fiber is demonstrated. The sensor consists of a gold film coated side-polished fiber covered by a layer of UV-curable adhesive. Before introducing the UV-curable adhesive, the gold-coated fiber exhibits refractive index (RI) sensitivity of 1691.6 nm/RIU to 8800 nm/RIU in the range of 1.32 to 1.43. The resonant wavelength of the SPR sensor shifts to 650 nm when the adhesive is coated on the gold film, and is fixed at about 725 nm when the adhesive is cured. Due to the high thermo-optic and thermal expansion coefficient of the adhesive, the sensor structure achieves a temperature sensitivity of −0.978 nm/°C between 25 °C and 100 °C. The proposed optical fiber SPR sensor is simple, highly sensitive and cost effective, which may find potential applications for temperature measurements in the biomedical and environmental industries.

## 1. Introduction

Over the past few decades, great attention has been paid to the surface plasmon resonance (SPR) sensors for its vital applications in chemical research and biosensing. SPR-based measurements of alcohol concentration [1], heavy metal ions [2,3], lipoprotein [4], pH [5,6], urea [7] and many chemical and biological analytes [8,9] have been reported in the literature. SPR generally occurs when the phase matching condition between the surface plasmon wave (SPW) of metal and incident electromagnetic wave [10] is satisfied. Earlier research of SPR mainly focused on the spatial prism structure. Optical fiber SPR sensors have become popular owing to numerous advantages, such as low cost, small tested volume, convenient operation and in vivo test capability. The reported configurations of optical fiber SPR sensors can be typically divided into two types [11]. The first type employs geometry-modified fibers, including unclad [12], tapered [13], side-polished, D-shaped [14], hetero-core structure [15] or U-shaped fibers [16], where the core-guided light is usually in direct contact with the surrounding medium. The second type is based on common fibers with embedded fiber gratings. The gratings here can be titled fiber Bragg gratings (tFBGs) [17] or long period fiber gratings (LPFGs) [18]. In these type of structures, the core-guided light can be diffracted into the cladding without destroying the mechanical integrity of the optical fiber. Fiber SPR sensors generally have higherrefractive index (RI) sensitivities compared with fiber grating or interferometer based RI sensors, and thus have been widely applied in many fields. Such advantages can be utilized to develop temperature sensors with high sensitivities.

A fiber SPR temperature sensor is usually formed by immersing a SPR RI sensor into a temperature sensitive medium. By transferring temperature variation to the RI change of medium, high temperature sensitivity can be achieved. Little work on temperature sensing with SPR-based optical fiber sensors has been reported so far, to the best of our knowledge. Zhao et al. reported a multimode fiber (MMF) SPR temperature sensor in 2015 by packaging a typical silver-coated MMF SPR sensor into a capillary filled with thermo-sensitive anhydrous ethanol, which exhibits a sensitivity of 1.5745 nm/°C [19]. Such a structure faces the difficulty of encapsulating alcohol, which makes the fabrication process complicated. Wang et al. reported a SPR temperature sensor based on a gold-polydimethylsiloxane (PDMS) coated MMF-Photonic Crystal fiber (PCF)-MMF structure [20], the temperature sensitivity of which was −1.551 nm/°C. While the sensitivity is high, the high full width at half maximum (FWHM) of the SPR spectrum actually limits the resolution of the sensor. Recently, Zhu et al. reported a single-mode twin-core fiber SPR temperature sensor by coating a layer of PDMS on the gold surface of the fiber SPR device. The sensor can be operated in the reflection mode with higher temperature sensitivities of 4.13 nm/°C to 2.07 nm/°C in the range of 20 °C to 70 °C [21]. In this paper, we proposed a simple optical fiber SPR temperature sensor. A single mode fiber is side-polished and coated with a layer of gold film at the flat surface to form a SPR structure. The SPR sensor exhibits RI sensitivity of 1691.6 nm/RIU to 8800 nm/RIU in the range of 1.32 to 1.43, and the maximum figure of merit (FOM) is 57/RIU. After coating the gold film with UV-cured adhesive, the resonance wavelength of the device shifted to 725 nm. A linear wavelength response to rising temperature is observed, and a sensitivity of −0.978 nm/°C is experimentally achieved from 25 °C to 100 °C. The proposed optical fiber SPR sensor is simple, highly sensitive, cost effective, and has improved mechanical strength due to the adhesive coating on the side-polished fiber. Such a device has potential applications in biomedical and environmental industries.

## 2. Device Fabrication and RI Sensing Characterization

**Figure 1:**
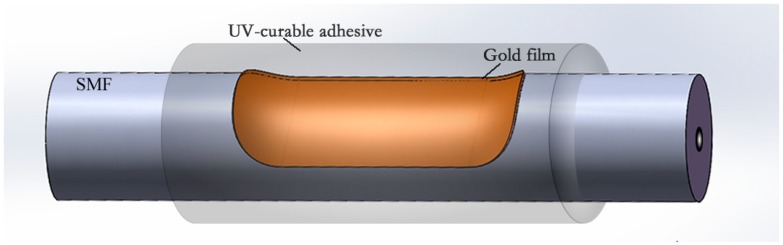
Schematic diagram of the proposed optical fiber surface plasmon resonance (SPR) temperature sensor.

**Figure 2:**
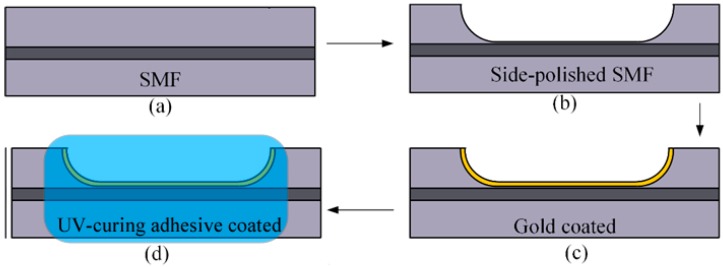
Schematic illustration of the fabrication process of the proposed sensor: (**a**) The original single mode fiber. (**b**) Side-polishing of the SMF. (**c**) Gold coating. (**d**) UV-curing adhesive coating.

Figure 1 shows the schematic of the proposed optical fiber SPR temperature sensor. A layer of low-index UV-curable adhesive is coated to the surface of the gold layer of a side-polished fiber SPR sensor. When the wave vector of the incident light in the fiber core and the SPW on the interface between gold and UV-curable satisfy the phase matching condition, typical resonant coupling occurs, and the transmission spectra in the output experience energy loss at particular wavelengths, which can be observed in the transmission spectrum. The fabrication process is illustrated in Figure 2. A standard single mode fiber (Corning SMF-28) with a core/cladding diameter of 8.2/125 μm was employed in the experiment, and the cladding was polished by the wheel polishing equipment described in our previous work [22]. In this work, the polished length and thickness of the residual fiber was about 5 mm and 70 μm, respectively. The side-polished fiber was first cleaned with acetone followed by washing with de-ionized water 3–5 times. Then, a layer of gold (Au) film of ~55 nm was coated to the flat surface of the side-polished fiber by magnetron sputtering technique, where the pressure of vacuum chamber is 5.5 × 10^−4^ Pa and the thickness of the gold film were measured by step measuring instruments. After the gold film was coated onto the side-polished fiber, typical SPR coupling can be observed in the transmission spectrum by immersing it into RI-matching liquids.

**Figure 3:**
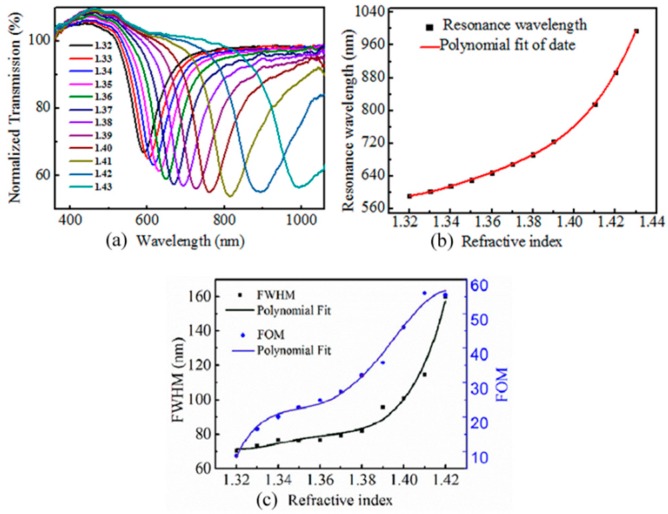
Refractive index (RI) test of SPR sensor before coating UV-curing adhesive. (**a**) Transmission spectra at different RIs. (**b**) SPR wavelength versus RI of the liquids; (**c**) Full width at half maximum (FWHM) and figure of merit (FOM) versus RI.

Before coating the UV-curable adhesive layer to the gold surface, the RI response of the fiber SPR device was investigated. The RI sensor was immersed into a series of refractive index matching liquids (Cargille Labs) with an index range of 1.320 to 1.430, and the transmission spectrum is recorded accordingly. The relationship between resonant wavelength and RI is given in Table 1, and the results are shown in Figure 3. From Figure 3a we can see that the resonant wavelength shifts towards longer wavelengths as RI increases, and the relationship between the resonant wavelength and surrounding RI is plotted in Figure 3b. The sensitivity increases with the value of RI, and reaches from 1691.6 nm/RIU at 1.330 to 8800 nm/RIU at 1.430. FOM is a parameter to evaluate the performance of the sensor, which is defined as the ratio of the sensitivity to the full width at half maximum (FWHM) of the transmission dip,

(1) $$\mathrm{FOM}=\frac{\mathrm{sensitivity}}{\mathrm{FWHM}}$$

The response of the FWHM and FOM against surrounding RIs are plotted in Figure 3c, marked as black solid squares and blue solid circles, respectively. The FWHM of the sensor broadens with the increase of RI when the value of RI increases, and the maximum FOM is 57/RIU at index value of 1.42. The resolution of the sensing device can be calculated by R = σ/S, where σ is the smallest measurement wavelength and S is the sensitivity. σ can be given by

(2) $$\sigma =\frac{\mathrm{FWHM}}{4.5\times \sqrt[{4}]{\mathrm{SNR}}}$$

In our case, FWHM varies from 70 nm to 160 nm, and the resolution for RI measurement is calculated to be in the range of 0.0017 to 0.004 RIU.

## 3. Temperature Sensing

**Figure 4:**
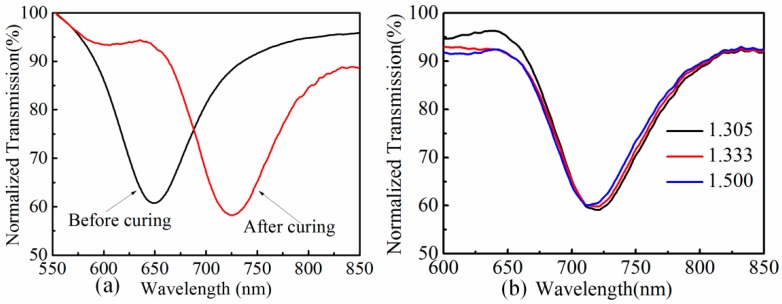
(**a**) Transmission spectra at different states of UV-curing adhesive; (**b**) RI test of optical fiber SPR sensor after coating with UV-curing adhesive.

**Figure 5:**
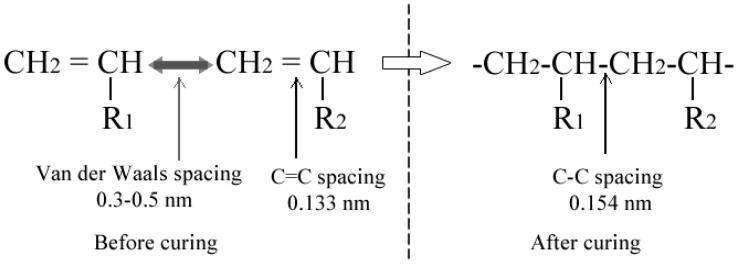
The change of atom spacing during curing process of UV-curable adhesive.

For the temperature sensing, the gold-coated fiber was dipped in a UV-curable adhesive (NOA 136, Norland Products, Inc) and then exposed to UV light for 5 min to ensure the adhesive is cured. The RI of the UV-curable adhesive is 1.36 before cured and the energy density required for full curing is ~6 J/cm^2^. When the uncured UV-curing adhesive is coated on the gold layer, the transmission spectra are shown in Figure 4a as a black solid line, where the resonant dip is at 649.4 nm, corresponding to the RI value of 1.36 from Table 1. After the curing process, the resonant dip shifts to 725.6 nm, which corresponds to the RI value of 1.39. This indicates that the RI of the UV-curing adhesive changes from 1.36 to 1.39 during the curing process. The increase of the RI can be explained by the volume shrinkage of the UV-curing adhesive, which originates from the shortening of inter-atomic spacing. The main constituents of the adhesive isacrylic, and the link between acrylate monomer molecules has changed from van der Waals forces to covalently linked during the polymerization; the spacing of the former is 0.3–0.5 nm, while that of the later is about 0.15 nm, as shown in Figure 5 [23]. The density of the adhesive increases as the molecules gets closer, which causes the refractive index to rise.

To test the RI response of the adhesive-coated sensor, the device was immersed in liquids with RI of 1.305, 1.333, and 1.500, and the spectrum for each case is shown in Figure 4b. The resonant wavelength changes very slightly as the surrounding medium RI increases. The introduction of UV-curing adhesive on the gold film has actually isolated the SPR structure from the outside medium, and the SPR wave can hardly be affected by the surrounding RI change. However, the changes of RI of the UV-curing adhesive layer still has a significant impact on the resonance wavelength of the SPR sensor. Therefore, when the surrounding temperature changes, the RI of the UV-curing adhesive layer changes fast due to the overall contribution of thermal expansion and thermal-optic effect, which leads to the shift of the resonant wavelength.

**Figure 6:**
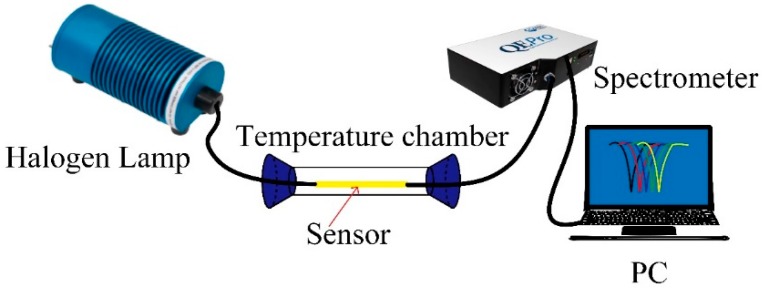
Schematic illustration of the temperature experiment system.

**Figure 7:**
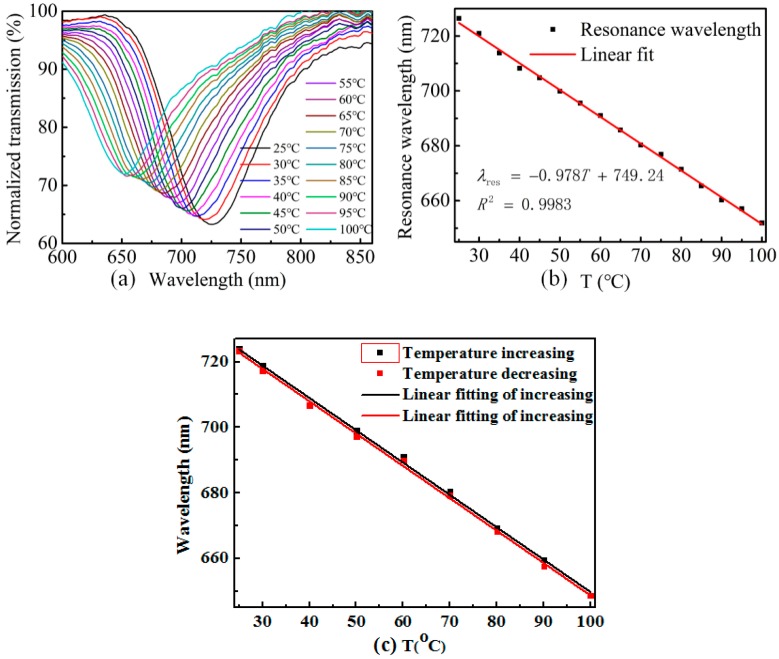
(**a**) Normalized transmission spectra of the sensor at different temperatures. (**b**) SPR wavelength versus the applied temperature. (**c**) The cycle experiment to characterize the reversibility of the sensor when temperature is increasing and decreasing.

The temperature response of the sensor is investigated by employing a heat treatment to the sensor with a tube furnace, the accuracy of which is ±0.1 °C and the measurement range is from room temperature to 100 °C. Figure 6 illustrates the schematic diagram of the equipment we use for temperature measurement. Un-polarized light from a halogen lamp is launched into the optical fiber SPR temperature sensor, and the transmitted signal is collected by a fiber-optic spectrometer (QE6500, Ocean Optics, Dunedin, FL, USA). The dip wavelength of the adhesive-coated optical fiber SPR sensor at room temperature (~25 °C) is ~725 nm. Each temperature point is maintained for about 10 min to obtain a stable transmission spectrum. The dip wavelength in transmission spectrum exhibits blue shift when the applied temperature is increased from 25 °C to 100 °C (below the glass transition temperature of the adhesive ~120 °C), as shown in Figure 7a. The resonant wavelengths versus temperature are plotted in Figure 7b, and a linear fitting was employed, from which we can see the averaged temperature sensitivity is −0.978 nm/°C with a correlation coefficient of R^2^ = 0.9983.

Since the reversibility of a fiber temperature sensor is an important factor that affects the performance in practical application, we fabricated another sample to investigate the reversibility of the sensor. As shown in Figure 7c, the wavelength dependence of the resonant wavelength on the temperature is measured in a heating/cooling cycle experiment. The temperature test is conducted in a humidity-controlled oven, where the relative humidity is kept at 30% during the test to avoid influence of humidity on the adhesive [24,25]. It is shown in Figure 7c that the black squares for the heating experiment overlapped with the red squares (cooling experiment) very well, which indicates the sensor has a good reversibility. As has been suggested above, the wavelength is caused by both the thermal-optic and thermal expansion effect. The thermal expansion effect causes the density of the adhesive to drop under rising temperature, while density is related to the RI by Lorentz–Lorenz law [26],

(n^2^ − 1)/(n^2^ + 2) = *ρ*RD/M (3)

where RD is molar refraction, M is molecular weight, and *ρ* is the density of the material. RD and M are constant under changing temperatures. In this way, the RI of the UV-cured adhesive decreases with density *ρ* when temperature increases, according to Equation (3).

Apart from the RI changing of the adhesive, the gold layer is also affected by the change of temperature [27,28]. The complex permittivity of gold layer can be described as [29],

(4) $$\varepsilon_{\mathrm{metal}}\left(T\right)=\varepsilon_{\infty }-\frac{\omega_{d}{\left(T\right)}^{2}}{\omega^{2}-{\omega \gamma }_{d}\left(T\right)i}$$

ε_metal_ represents the permittivity of the metal, ε_∞_ is the permittivity in high frequency, ω_d_(T) and γ_d_(T) are the plasma frequency and damping frequency, respectively. Temperature has an influence on both ω_d_(T) and γ_d_(T), which causes the SPR wavelength to change. Simulation results indicate that the temperature sensitivity of a gold-coated side-polished SPR sensor (gold layer thickness of 42 nm, and polished fiber residual thickness of 68 µm) under fixed ambient RI (1.33) is about 0.13 nm/°C [27]. So for our case, the wavelength shift of the polymer coated SPR sensor under rising temperature is resulting from the overall effect of the blue shift caused by the adhesive, and the red shift caused by the gold layer. From the experimental results, we can tell that the resonant wavelength shift from 725 nm to 651.6 nm when temperature rises from 25 °C to 100 °C, which correspond to the RI of the adhesive changing from 1.39 to 1.36 in Figure 3b, and the thermo-optic coefficient of the adhesive can be calculated to be 4 × 10^−4^/°C. This value is close to that of some acrylic based adhesives [26]. However, considering the red shift of wavelength caused by the temperature influence on the gold layer (about 10 nm), the RI of the adhesive should have changed more to compensate the red shift, and thus the thermo-optic coefficient should be even higher.

**Figure 8:**
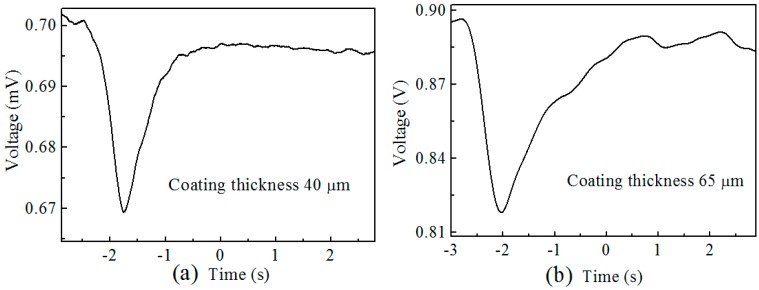
Response time of samples with UV cured adhesive coating with thickness of (**a**) 40 µm and (**b**) 65 µm.

The response time of the sensor is experimentally investigated. A laser diode with a wavelength of 660 nm is used to illuminate the sensor, and the output signal is collected by a fast response photo-detector, which is connected to an oscilloscope. The sensor structure is heated rapidly by using CO_2_ laser illumination, and a quick drop in voltage is observed. Two samples with different coating thickness (40 µm and 65 µm) are tested for the time response, and the response time is approximately 500 ms for both devices, as can be seen from Figure 8. The returning time is longer for the 65 µm device. The coating thickness may not be precisely the same in the fabrication of different samples, but can be controlled in certain ranges to achieve a stable response time.

## 4. Conclusions

A novel structure of a fiber optic SPR temperature sensor was proposed and experimentally demonstrated. The SPR sensor exhibits a RI sensitivity of 1691.6 nm/RIU to 8800 nm/RIU in the range of 1.32 to 1.43, and the maximum FOM is 57/RIU. After coating the gold film with UV-cured adhesive, a linear wavelength response to rising temperature is observed, and a sensitivity of −0.978 nm/°C is experimentally achieved from 25 °C to 100 °C. The proposed optical fiber SPR sensor is simple, highly sensitive and cost effective, and has potential applications in biomedical and environmental industries.

## Figures and Tables

**Table 1:** The relationship between the resonance wavelength and RI.

RI	1.32	1.33	1.34	1.35	1.36	1.37	1.38	1.39	1.40	1.41	1.42	1.43
Λres (nm)	591.5	602.5	616.2	630.9	648.3	669.0	693.0	725.4	761.0	815.3	893.7	995.5
